# Primary vaginal yolk sac tumor in an 11-month-old girl: a case report

**DOI:** 10.3389/fonc.2026.1759003

**Published:** 2026-03-05

**Authors:** Jiacheng You, Wei Xia

**Affiliations:** 1 Department of Radiology, Wuhan Children‘s Hospital Graduate Joint Training Base, School of Medicine, Wuhan University of Science and Technology, Wuhan, China; 2 Wuhan Children's Hospital (Wuhan Maternal and Child Healthcare Hospital), Tongji Medical College, Huazhong University of Science and Technology, Wuhan, China

**Keywords:** case report, extragonadal, gynecology, pediatrics, yolk sac tumor

## Abstract

**Background:**

Extragonadal yolk sac tumors are exceedingly rare. This report describes a vaginal yolk sac tumor in an infant to highlight key diagnostic and therapeutic aspects and its inclusion in the differential diagnoses of unexplained vaginal masses.

**Case presentation:**

An 11-month-old girl presented with intermittent vaginal bleeding, initially misdiagnosed as a hematoma. Pathological examination confirmed yolk sac tumors. She underwent hysteroscopic resection and six cycles of PEB (bleomycin, etoposide, cisplatin) chemotherapy. No recurrence occurred during the 12-month follow-up.

**Conclusion:**

Primary vaginal yolk sac tumors are extremely rare and lack standardized treatment. Surgery combined with PEB chemotherapy proved effective in this case.

## Introduction

Vaginal yolk sac tumor (VYST) is a highly uncommon malignant neoplasm. Although yolk sac tumors (YST) typically originate in the gonads, they may also arise in extragonadal sites such as the sacrococcygeal region, cervix, retroperitoneum, and intracranial cavity (1, 2). Diagnosing extragonadal YST is challenging. The cornerstone of diagnosis remains comprehensive histopathological examination of the tissue, with identification of characteristic morphological features such as Schiller-Duval bodies. This morphological assessment is powerfully supplemented by immunohistochemical profiling, which is an integral part of contemporary pathological evaluation. The diagnostic workup is further supported by a multimodal clinical approach that includes serum tumor marker assays, notably alpha-fetoprotein (AFP) (3, 4). Early recognition and intervention are crucial for improving prognosis. Notably, genital tract tumors account for 2%–21% of prepubertal vaginal bleeding cases, underscoring the necessity of including malignancy in the differential diagnosis (5). We report a rare case of VYST presenting solely with vaginal bleeding and discuss its early diagnosis and management.

## Case presentation

### Preoperative condition

On June 9, 2024 (Day 1), an 11-month-old girl was referred to the gynecologic outpatient clinic of our institution with a one-month history of intermittent vaginal bleeding. Physical examination revealed normal prepubertal genital development. The infant was born at term without perinatal complications and had no family history of malignancy. Initial transvaginal ultrasound, performed while the child was agitated, showed ambiguous vascular signals within the lesion, leading to a preliminary diagnosis of a vaginal hematoma. However, the consulting gynecologist questioned this diagnosis based on clinical suspicion.

**Figure 1:**
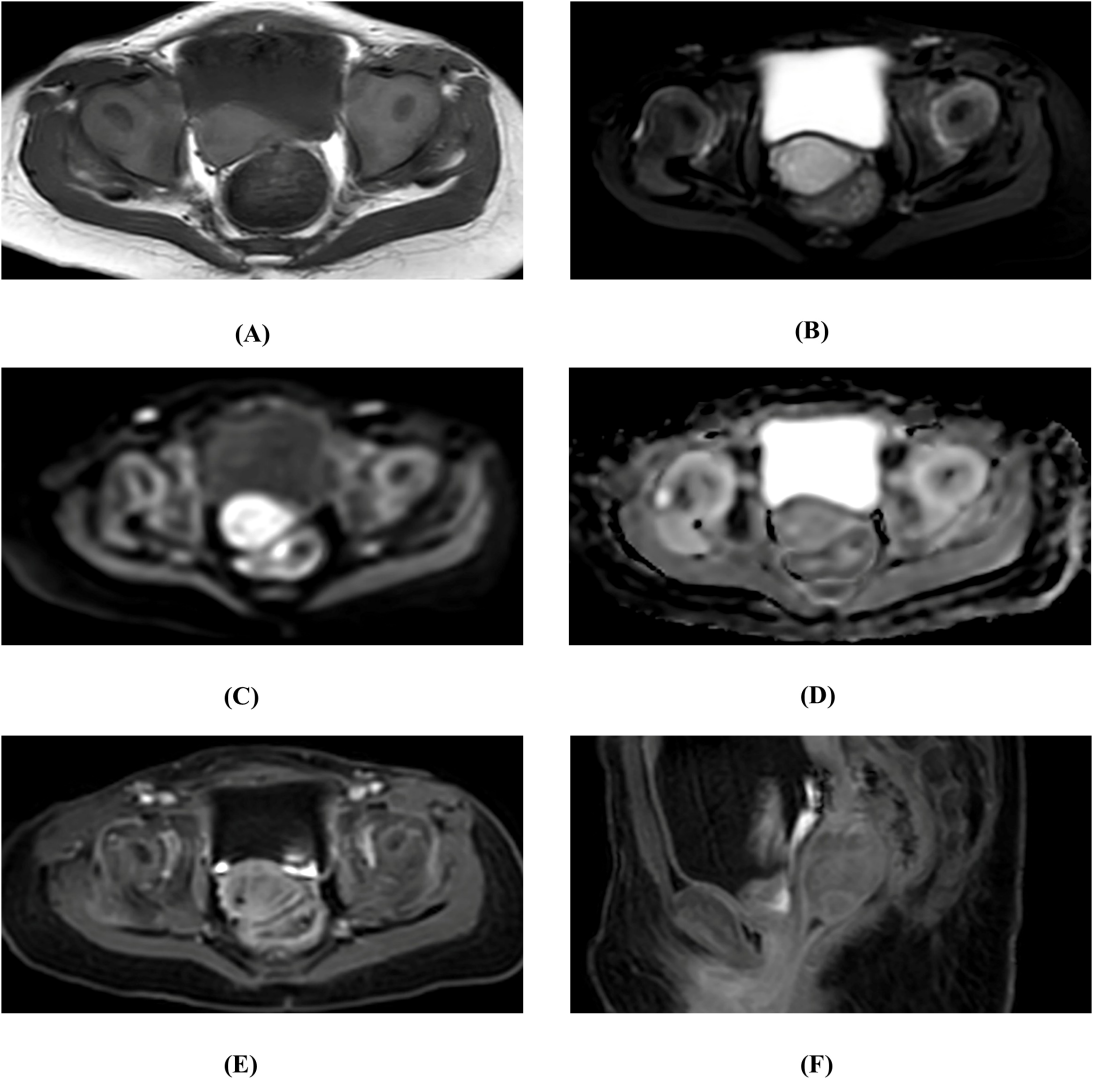
The results of the preoperative examination. **(A)** Axial T1-weighted image (T1WI). **(B)** Axial fat-suppressed T2-weighted image (FS T2WI). **(C)** Axial diffusion-weighted image (DWI; b = 800 mm^2^/s). **(D)** Axial apparent diffusion coefficient **(ADC)** map. **(E)** Axial post-contrast fat-suppressed T1WI. **(F)** Sagittal post-contrast fat-suppressed T1WI. Fat-suppressed T2WI shows a vaginal tumor with slightly heterogeneous high signal intensity. On high-b-value DWI, the solid-enhancing part of the tumor exhibits high signal intensity, with a low ADC (ADC mean = 1.2×10–^3^ mm^2^/s). The mass shows mild to moderate enhancement on post-contrast fat-suppressed T1WI, with enhancement lower than that of the adjacent vaginal tissue.

To obtain a definitive diagnosis and avoid misdiagnosis, the patient was admitted for further evaluation on the same day. On June 10, 2024 (Day 2), pelvic magnetic resonance imaging (MRI) revealed a well-defined mass in the mid- to upper vagina measuring approximately 22 mm × 16 mm × 20 mm. The lesion exhibited mild hyperintensity on T1-weighted imaging (T1WI), heterogeneous hyperintensity on T2-weighted imaging (T2WI), restricted diffusion on high b-value diffusion-weighted imaging (DWI), and corresponding low signal on the apparent diffusion coefficient (ADC) map. Post-contrast fat-suppressed T1-weighted sequences demonstrated heterogeneous enhancement of the mass. No significant abnormalities were observed in the uterus or adnexa, and there was no evidence of pelvic lymphadenopathy or ascites (**Figures 1A–F**). Vaginoscopy was not performed to avoid the risk of iatrogenic injury to prepubertal tissues. Concurrent serological tests showed a markedly elevated alpha-fetoprotein level of 1647.3 ng/ml (reference <7 ng/ml), while carcinoembryonic antigen (CEA), carbohydrate antigens (CA125, CA19-9), beta-human chorionic gonadotropin (β-hCG), and sex hormones were all within normal ranges.

The preoperative diagnosis was formulated through a comprehensive synthesis of clinical, laboratory, and imaging findings. Endocrine disorders such as precocious puberty were considered unlikely given the patient’s age, absence of breast or pubic hair development, lack of café-au-lait spots, and no history of exogenous hormone exposure. Traumatic etiology was also excluded based on the absence of a reported injury history, along with ultrasonographic findings showing no soft tissue swelling or foreign body adjacent to the lesion. The intermittent vaginal bleeding, in conjunction with a well-defined solid mass localized to the vagina on MRI and a markedly elevated serum alpha-fetoprotein (AFP) level of 1647.3 ng/ml—a highly specific biomarker for yolk sac tumors in this age group—collectively provided a compelling rationale for diagnostic surgical intervention. The diagnosis was subsequently confirmed by postoperative histopathological examination.

### Operative condition and pathologic results

**Figure 2:**
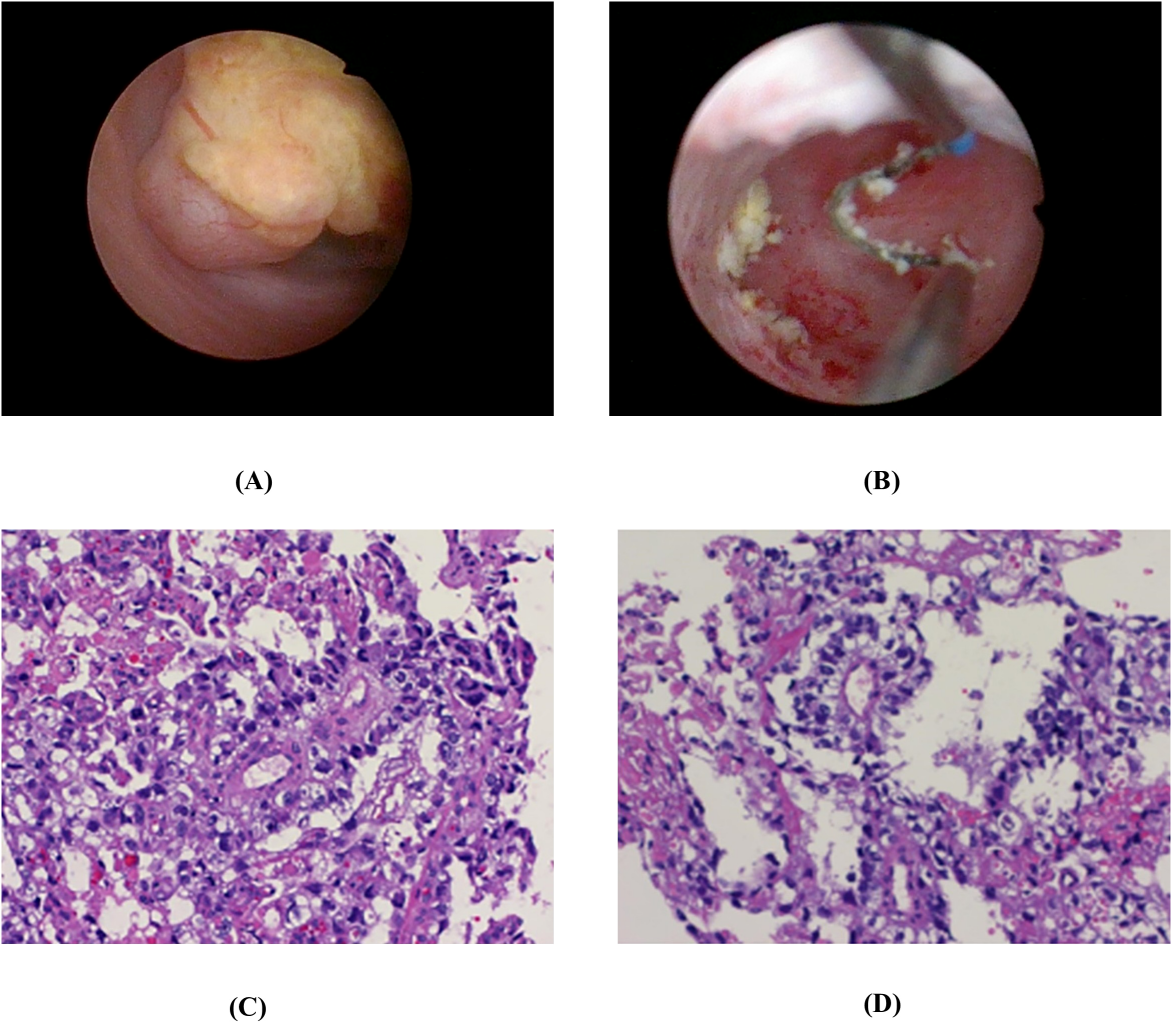
Hysteroscopic and microscopic findings of the vaginal yolk sac tumor. **(A)** Pre-resection hysteroscopic view reveals a yellowish, irregular mass with a narrow stalk located on the right posterior vaginal wall. **(B)** Post-resection hysteroscopic view shows a pale yellow residual tumor stalk at the vaginal wall wound. **(C)** Diagnostic Schiller–Duval body. Original magnification, ×200. **(D)** A central vessel surrounded by a layer of tumor cells. Original magnification, ×200.

Following preoperative evaluation, the patient underwent hysteroscopic resection on June 14, 2024. Hysteroscopy revealed a yellowish, friable, irregular mass measuring approximately 30 mm × 25 mm × 10 mm with a narrow stalk, located on the right posterior vaginal wall (**Figure 2A**). To preserve vaginal anatomy and the infant’s future reproductive potential, a conservative surgical approach was adopted. This involved resection of the gross tumor mass with minimal margins of adjacent vaginal tissue, without performing a radical resection (**Figure 2B**). Postoperative imaging confirmed that the tumor was confined to the vaginal wall with no extra-vaginal extension.

Histopathological examination of the postoperative specimen revealed diagnostic Schiller-Duval bodies accompanied by microcystic and glandular patterns with significant nuclear atypia (**Figures 2C, D**). Immunohistochemical analysis confirmed the diagnosis, demonstrating positivity for AFP, Glypican-3, CD117, placental alkaline phosphatase (PLAP), Sal-like protein 4 (SALL4), and Cytokeratin (CK), with a Ki-67 index of 70%. According to the Children’s Oncology Group (COG) criteria, the tumor was classified as Stage III (high-risk group).

### Postoperative chemotherapy and prognosis condition

**Figure 3:**
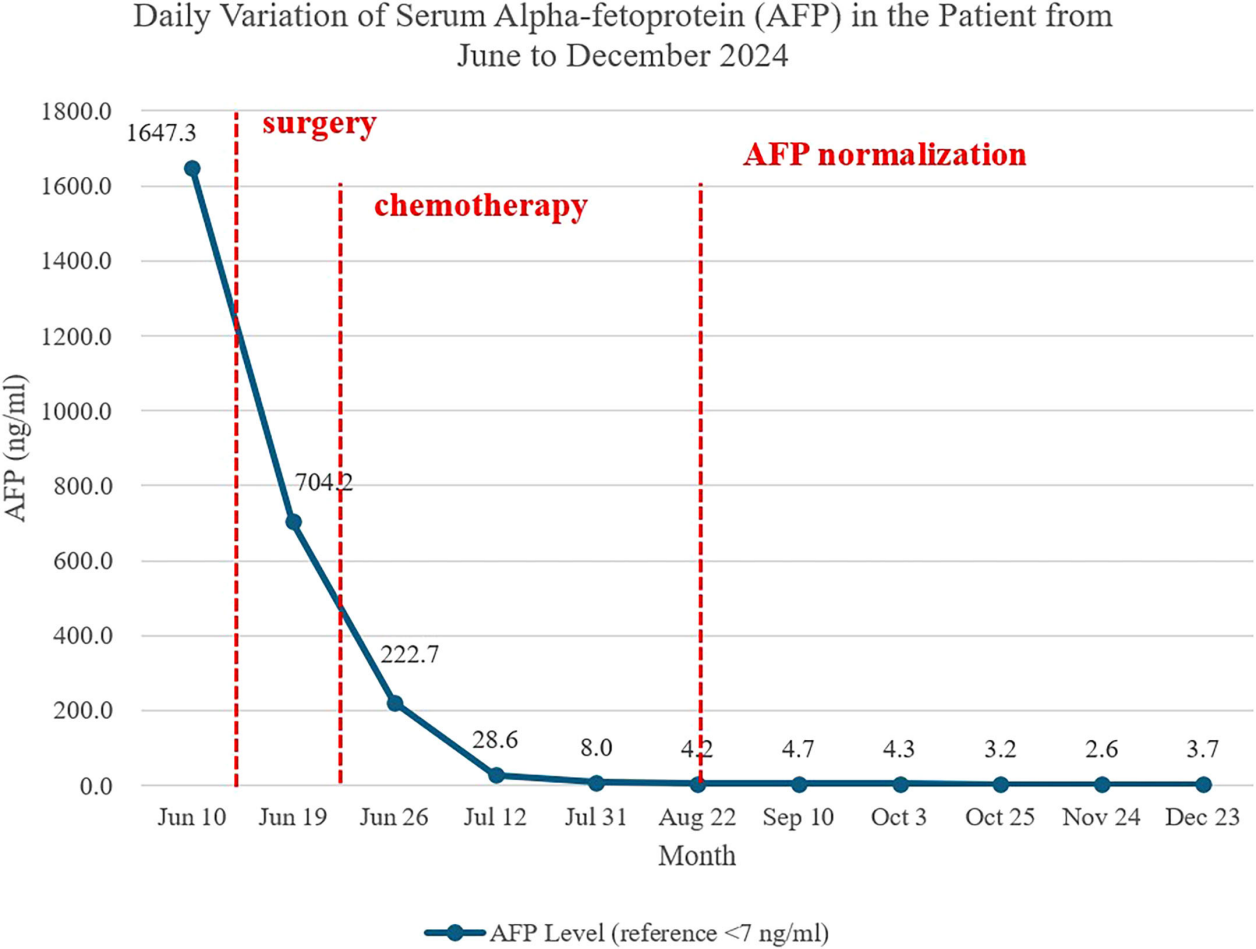
The results of postoperative reexamination. Daily monitored values of serum alpha-fetoprotein (AFP) concentration in the pediatric patient from June 1 to December 31, 2024. The line illustrates the dynamic changes in AFP levels (unit: ng/ml). The patient underwent vaginal lesion resection on June 14, 2024, and received six cycles of chemotherapy on June 22-26, July 12-16, August 2-6, August 23-27, September 13-17, and October 4-8, 2024.

The patient was transferred to the pediatric oncology ward on June 18, 2024, for further management. Comprehensive staging evaluation via computed tomography (CT), MRI, and whole-body bone scintigraphy showed no evidence of residual or metastatic disease. On June 19 (postoperative day 5), the serum AFP level had significantly decreased to 704.2 ng/ml. With the initiation of adjuvant PEB chemotherapy (cisplatin, etoposide, bleomycin), the AFP level continued to decline rapidly. It normalized to 4.2 ng/ml after completion of the third chemotherapy cycle (postoperative day 69), achieving a biochemical complete remission (**Figure 3**). She subsequently completed the next three cycles of PEB chemotherapy, totaling six cycles, administered according to the pediatric protocol for high-risk germ cell tumors, with cycles repeated every 21 days. Her AFP level has remained within the normal range thereafter. During the 12-month follow-up period, serial AFP measurements and MRI examinations showed no evidence of tumor recurrence or residual disease. The timeline of the clinical management is illustrated in **Table 1**.

**Table 1:** The flowchart of timeline for diagnosis and treatment process.

Time point	Event description	Key actions/Findings
Before symptom onset	No significant medical history	None
May 2024	Intermittent vaginal bleeding occurred twice	None
June 9, 2024	Initial visit to the gynecologic outpatient clinic	Physical examination revealed only blood staining at the vaginal introitus. Initial ultrasound led to a misdiagnosis of vaginal hematoma.
June 10, 2024	Further imaging evaluation	Pelvic MRI identified a vaginal mass.
Same day	Tumor marker assessment	Serum AFP level was 1647.3 ng/ml.
June 14, 2024	Preoperative evaluation and surgery	Underwent hysteroscopic resection.
Same day	Examination of the surgical specimen	Pathological examination confirmed the diagnosis of vaginal yolk sac tumor.
June 18, 2024	Transferred to the oncology department for treatment, along with postoperative staging and metastasis evaluation	CT, MRI, and whole-body bone scintigraphy showed no evidence of metastasis.
June 19, 2024	Follow-up AFP test	The AFP level had significantly decreased to 704.2 ng/ml
June 22, 2024	Initiation of chemotherapy with concurrent AFP monitoring	Received six cycles of PEB chemotherapy (cisplatin, etoposide, bleomycin), accompanied by a significant decrease in AFP levels.
August 22, 2024	Follow-up AFP test	Completed three cycles of chemotherapy, with AFP levels within the normal range.
From October 8, 2024, to present	Completion of chemotherapy and entry into the follow-up phase	Regular AFP monitoring and imaging surveillance showed no evidence of tumor metastasis or recurrence.

## Discussion

### Epidemiological and clinical characteristics

Vaginal tumors are uncommon across all age groups, with most cases being malignant. Subtypes exhibit age-related variation: squamous cell carcinoma predominates in adults, while rhabdomyosarcoma is the most common pediatric malignant vaginal tumor, typically presenting as a “grape-like” mass. VYST is among the rarest malignant entities, with only approximately 100 cases documented in the medical literature to date (6).

YST is a rare malignant germ cell neoplasm with well-defined epidemiological characteristics. It represents the third most common histologic subtype among ovarian malignant germ cell tumors (OMGCTs), accounting for approximately 14.5% to 16.4% of all OMGCTs (7). While most frequently occurring in the gonads, extragonadal presentations of YST are exceptionally rare, with an estimated incidence of 1.8–3.4 cases per million population (8). YSTs predominantly affect children, adolescents, and young adults, with a median age at diagnosis of approximately 18 years. However, VYST constitutes a specific and rare extragonadal variant, occurring almost exclusively in girls under 3 years of age. The clinical presentation in these patients is non-specific, most commonly manifesting as vaginal bleeding or blood-tinged discharge, and occasionally as a palpable mass (9). A hallmark epidemiological feature of YST, irrespective of the primary site, is the marked elevation of AFP in the vast majority of cases, which underpins its pivotal role in diagnosis and clinical management.

The patient described herein presented with vaginal bleeding alone before hospitalization. This symptom is uncommon in non-verbal toddlers. Epidemiological data indicate that benign etiologies dominate the differential diagnosis, with vulvovaginitis being the most common cause, followed by vaginal foreign bodies, urethral prolapse, and trauma. Nevertheless, neoplastic causes must be considered, with RMS being the most common malignant cause of infant vaginal bleeding (10, 11).

### Imaging and differential diagnosis

Ultrasound is often employed as a first-line imaging modality due to its accessibility, low cost, and non-invasive nature. In this case, the patient presented with a one-month history of intermittent vaginal bleeding. The ultrasound report described the lesion as having relatively homogeneous echogenicity with detectable, though indistinct, vascular signals on Doppler imaging. It is well established that acute or subacute hematomas typically exhibit heterogeneous internal echogenicity and lack internal vascularity on color Doppler—a key feature distinguishing them from vascular or malignant tumors. Exceptions may occur during the organizing phase of a hematoma (2–3 weeks post-injury), when ingrowth of nascent capillaries may give rise to faint punctate blood flow signals. Similarly, transient signals may be detected during active bleeding, although these are seldom captured under ultrasound imaging (12). The initial misdiagnosis likely resulted from a combination of these uncommon scenarios and the limited resolution of ultrasound. Subsequent non-enhanced and enhanced MRIs demonstrated relatively homogeneous signal intensity, inconsistent with the heterogeneous signal pattern typically seen in a complex hematoma. Moreover, the mass exhibited heterogeneous enhancement, further supporting the diagnosis of a neoplastic lesion rather than a hematoma.

Imaging plays a crucial role in the preoperative assessment of suspected YST. Although manifestations vary with the primary site, common features exist across different modalities. Ultrasonography may reveal a complex solid-cystic mass with internal vascular flow. CT typically shows a heterogeneously enhancing soft-tissue mass. MRI, by virtue of its superior soft-tissue contrast, is the preferred modality for local staging and characterization. YSTs typically present as well-demarcated masses exhibiting heterogeneous high signal intensity on T2-weighted images—a reflection of their mixed solid, microcystic, and occasionally hemorrhagic composition. Post-contrast sequences demonstrate moderate-to-marked heterogeneous enhancement of the solid components (13). In the present case, these characteristic MRI findings were clearly evident.

MRI is particularly valuable in distinguishing YST from other conditions in the differential diagnosis of pediatric pelvic masses. Compared to non-neoplastic lesions such as hematomas, the key distinction lies in the latter’s lack of enhancing tissue and its predictable evolution of signal intensity. For differentiation from other malignant tumors, the following points are helpful: (A) Rhabdomyosarcoma, the most common malignant vaginal tumor in children, often presents with a multilobulated, infiltrative (“grape-like” or botryoid) morphology, contrasting with the more circumscribed appearance of YST. (B) Teratomas contain macroscopic fat and/or calcification, elements absent in pure YST. (C) Other malignant germ cell tumors may exhibit more homogeneous enhancement and are not associated with profoundly elevated AFP. It is paramount to recognize that these imaging patterns are not unique; therefore, their interpretation must be integrated with the serum tumor marker profile (14, 15). The combination of a solid, heterogeneously enhancing mass on MRI with a significantly elevated serum AFP level is highly specific for YST and formed the basis for the preoperative diagnosis in this case.

### Histopathology and differential diagnosis

YST originates from primordial germ cells and exhibits a spectrum of histological patterns, including reticular-microcystic, glandular, solid, papillary, and hepatoid variants. The most characteristic morphology is the reticular-microcystic pattern, characterized by an anastomosing network of microcysts and channels lined by flattened or cuboidal tumor cells with pale to clear cytoplasm, set within a myxoid stroma (16). A pathognomonic structure, the Schiller-Duval body, may be present, featuring a central fibrovascular core surrounded by radially arranged tumor cells, recapitulating the endodermal sinus pattern of the rodent placenta—hence the synonym endodermal sinus tumor (EST). However, these diagnostic structures are identified in only approximately 20%–30% of cases. Therefore, their absence does not preclude the diagnosis. Definitive diagnosis often requires integration with a supportive immunohistochemical profile, typically demonstrating positivity for AFP and glypican-3, alongside an elevated Ki-67 proliferation index (17, 18). This combined morphological and immunophenotypic approach is crucial for differentiating YST from other germ cell neoplasms.

Key differential diagnoses include dysgerminoma and seminoma, which display sheets or nests of large, uniform cells with clear cytoplasm, central nuclei, prominent nucleoli, and a characteristic lymphocytic infiltrate within fibrous septa. It lacks the reticular-microcystic architecture and Schiller-Duval bodies of YST and typically expresses OCT3/4, PLAP, and CD117, with absent or weak expression of AFP and glypican-3. Immature teratoma is defined by the presence of immature neuroectodermal elements, often with other immature or mature tissues from multiple germ layers, a feature absent in YST. Immunohistochemically, its immature components express markers like GFAP and NSE but not specific germ cell tumor markers. Embryonal carcinoma exhibits solid, glandular, or papillary growth of highly pleomorphic cells and eosinophilic cytoplasm, frequently associated with hemorrhage and necrosis. It is characterized by expression of CD30 and OCT3/4. Choriocarcinoma is composed of a biphasic proliferation of cytotrophoblast and syncytiotrophoblast cells in a plexiform pattern and is characterized by HCG expression (19). In the present case, the coexistence of Schiller-Duval bodies and strong AFP immunoreactivity confirmed the diagnosis.

### Treatment and chemotherapy

Owing to its extreme rarity, no established treatment guidelines exist for VYST. Untreated cases progress rapidly, often culminating in death within 2–4 months, highlighting the critical importance of early intervention (20). Historically, treatment involved radical surgery with or without radiotherapy, resulting in poor outcomes and survival of approximately 2 years (21). Therefore, the Children’s Oncology Group (COG) has established a staging system for pediatric germ cell tumors that guides the selection of appropriate treatment strategies based on tumor type, location, and stage. For prepubertal patients with yolk sac tumors, Stage I disease is managed with surgical resection alone, while Stages II-IV require a combination of surgical resection and chemotherapy (22, 23). The patient in this case was classified as Stage III (high-risk group); accordingly, our institution adopted a combined surgical and chemotherapeutic approach in line with these treatment guidelines.

In recent years, the PEB chemotherapy regimen has gradually replaced the earlier VAC regimen (vincristine, actinomycin D, and cyclophosphamide) as the mainstream approach, leading to marked improvement in treatment outcomes for pediatric YST. Based on the adult BEP regimen, the Pediatric Oncology Group (POG) and the Children’s Cancer Group (CCG) developed a PEB protocol adapted for children by adjusting dosages. This adaptation is driven primarily by the imperative to maintain antitumor efficacy while reducing long-term toxicity to developing organs. The most significant modification lies in the administration of bleomycin. In the adult BEP regimen, bleomycin is typically administered once weekly (30 mg/m²) to intensify the therapeutic response. In contrast, the pediatric protocol, especially for children under 3 years of age, strictly limits bleomycin to a single dose per cycle (15 mg/m² on day 1). This substantial reduction aims to minimize cumulative exposure and the associated risk of life-threatening pulmonary fibrosis (24). Furthermore, dose adjustments for cisplatin and etoposide in infants must be guided by immature renal and hepatic function. This often involves weight-based calculations or the application of a reduced percentage (75%) of the standard pediatric dose calculated by body surface area for infants under one year of age. This meticulous and individualized dosing strategy aims to balance antitumor activity against the heightened risks of cisplatin-induced ototoxicity and nephrotoxicity, as well as the potential risk of etoposide-related secondary leukemia. The treatment typically consists of approximately 3 to 6 cycles administered at 21-day intervals, with therapeutic response monitored through serial AFP quantification (25). Consolidation chemotherapy continues for 2–3 cycles after AFP normalization. This approach has improved overall survival to approximately 90% while effectively preserving fertility (26).

Postoperatively, patients should undergo regular surveillance, including physical examination, serum AFP testing, and imaging, to confirm sustained complete remission (27, 28). Serial AFP quantification is paramount in the management of YST. It serves as a highly specific diagnostic adjunct, a sensitive real-time marker for assessing chemotherapeutic response, a guide for determining the need for consolidation therapy, and the most critical tool for the early detection of recurrence during long-term surveillance (11).

### Prognostic factors

The prognosis of YST is significantly influenced by well-defined clinicopathological factors, highlighting the importance of risk-adapted management. Clinical stage at diagnosis is the foremost prognostic determinant, with key assessment metrics including tumor stage, size, and the presence of distant metastasis. Advanced-stage disease—such as FIGO stage III/IV, tumor size >10 cm, or the presence of metastases—is consistently associated with a substantially elevated risk of postoperative recurrence. In vaginal YST, for instance, patients with disease confined to the vaginal wall (stage I) who receive PEB chemotherapy uniformly achieve complete remission, with a median recurrence-free survival of 53 months. In contrast, cases involving pelvic tissues (stage II or higher) exhibit a 2.3-fold increased recurrence risk despite intensive chemotherapy (29). Similarly, for central nervous system (CNS) YST, the 5-year overall survival rate drops to 38.5% when cerebrospinal fluid dissemination or distant metastasis is present (30). These findings collectively confirm that a more advanced clinical stage correlates with greater tumor aggressiveness and poorer prognosis, underscoring that early diagnosis and radical therapy are pivotal for improving outcomes.

Histological features also provide critical prognostic information. “Pure” YST generally demonstrates higher sensitivity to platinum-based chemotherapy. In contrast, mixed germ cell tumors that contain other malignant components—such as immature teratoma, embryonal carcinoma, or choriocarcinoma—show reduced 5-year survival rates (40%–50%), owing to the chemoresistance and enhanced aggressiveness of the heterogeneous elements. Furthermore, certain morphological subtypes (e.g., the hepatoid variant, which carries a worse prognosis than the classic reticular-microcystic pattern) and the presence of somatic malignant transformation are associated with increased chemoresistance and a higher risk of recurrence (31). Therefore, a comprehensive evaluation integrating both clinical stage and histological characteristics is essential for accurate prognostication and for tailoring the intensity of therapy.

## Strengths and limitations

This study provides a detailed account of a rare case of primary VYST in an 11-month-old infant, thereby enriching the scarce literature on this entity in early infancy. The report comprehensively describes the diagnostic pathway, underscoring the critical role of integrating pelvic MRI findings with serial serum AFP measurement in achieving a timely and accurate preoperative diagnosis. Furthermore, it explicitly outlines the clinical rationale for implementing fertility-preserving conservative surgery and a dose-adapted PEB chemotherapy regimen in a young child, which may inform management decisions in similarly challenging cases. The availability of complete clinical, imaging, and serological follow-up data over 12 months enhances the robustness of the presented clinical narrative.

The limitations of this work are inherent to its design as a single case report. The findings are descriptive and lack generalizability. While the follow-up period demonstrates favorable short-term oncological outcomes, it is insufficient for assessing long-term survival, late recurrence, and potential treatment-related sequelae, such as the impact of chemotherapy on future fertility, endocrine function, and the risk of secondary malignancies. Finally, the treatment approach was selected based on institutional protocol and consensus guidelines; this report does not provide comparative evidence for alternative therapeutic strategies.

## Conclusion

We present this VYST case to underscore key diagnostic imperatives. Although rare, VYST must be considered in the differential diagnosis of prepubertal vaginal bleeding. Timely serum tumor marker assessment (notably AFP) combined with pelvic MRI should be prioritized when etiology is unclear; if necessary, vaginoscopy and biopsy should be performed for early detection and treatment of the tumor. Histopathological confirmation—supported by characteristic morphology and immunohistochemical profiling—remains diagnostic. With early diagnosis and contemporary therapy, the prognosis for patients with VYST has improved significantly, with preservation of sexual and reproductive function largely achievable. However, it remains an aggressive neoplasm necessitating vigilant management.

## Patient perspective

The patient’s family consented to the treatment plan and expressed profound gratitude. The early and accurate diagnosis of vaginal yolk sac tumor at our institution enabled timely surgical intervention. Tumor resection effectively preserved the child’s reproductive potential, and the six cycles of PEB chemotherapy successfully prevented recurrence.

## Data Availability

The original contributions presented in the study are included in the article/supplementary material. Further inquiries can be directed to the corresponding author.
